# A practice guide on antimicrobial stewardship in nursing homes

**DOI:** 10.1186/s13756-023-01321-0

**Published:** 2023-11-03

**Authors:** Andrea Eikelenboom-Boskamp, Mariëlle van Loosbroek, Evelien Lutke-Schipholt, Marjorie Nelissen-Vrancken, Mike Verkaaik, Paul Geels, Stephanie Natsch, Andreas Voss

**Affiliations:** 1 Department of Medical Microbiology and Infectious Diseases, Canisius-Wilhelmina Hospital, Nijmegen, The Netherlands; 2 ZZG Care Group, Nijmegen, The Netherlands; 3 Department of Pharmacy, Canisius-Wilhelmina Hospital, Nijmegen, The Netherlands; 4 Dutch Institute for Rational Use of Medicine, Utrecht, The Netherlands; 5 The Dutch Working Party on Antibiotic Policy (SWAB), Leiden, The Netherlands; 6 Department of Pharmacy, Radboud University Medical Center, Nijmegen, The Netherlands; 7 Department of Medical Microbiology, Radboud University Medical Center, Nijmegen, The Netherlands; 8 Department of Medical Microbiology and Infection- prevention, University Medical Center Groningen, Groningen, The Netherlands; 9 Present Address: Knowledge Institute of the Dutch Association of Medical Specialists, Utrecht, The Netherlands

**Keywords:** Antimicrobial stewardship, Antibiotic team (A-team), Nursing homes, Practice guide

## Abstract

A practice guide to help nursing homes set up an antimicrobial stewardship (AMS) program was developed based on experiences gained during a project at one of the largest providers of elderly care in the South-east of the Netherlands. The guideline for the implementation of AMS in Dutch hospitals served as a starting point and were tailored to the unique characteristics of a nursing home setting. This practice guide offers recommendations and practical tools while emphasizing the importance of establishing a multidisciplinary approach to oversee AMS efforts. The recommendations and practical tools address various elements of AMS, including the basic conditions to initiate an AMS program and a comprehensive approach to embed an AMS program. This approach involves educating nurses and caregivers, informing volunteers and residents/their representatives, and the activities of an antibiotic team (A-team). The practice guide also highlights a feasible work process for the A-team. This process aims to achieve a culture of continuous learning and improvement that can enhance the overall quality of antibiotic prescribing rather than making individual adjustments to client prescriptions. Overall, this practice guide aims to help nursing homes establish an AMS program through collaborative efforts between involved physicians, pharmacists, clinical microbiologists, and infection control practitioners. The involved physician plays a crucial role in instilling a sense of urgency and developing a stepwise strategy.

## Introduction

Antimicrobial resistance is widely recognized as a crucial concern. Antibiotic use and antimicrobial resistance in long-term care facilities (LTCFs) are substantial due to the significant use of antibiotics [1]. Several studies have demonstrated high rates of inappropriate prescribing of antibiotics, reaching 24% or higher, in LTCFs [2–6]. Notably, in recent years, Dutch LTCFs have shown considerable variability in antibiotic use across facilities [7–11], with a recorded minimum of 2.1 and a maximum of 288.7 defined daily doses (DDD)/1,000 residents per day in 2021 [11].

Since 2015, it has been mandatory for Dutch hospitals to implement an antimicrobial stewardship (AMS) program to enhance the quality of antibiotic use. Consequently, a practice guide was developed to facilitate this process [12]. However, a ‘copy & paste’ approach to transfer hospital recommendations to nursing homes (NHs) was deemed unsuitable due to substantial differences in organizational structures between these two settings. The connections between electronic medical records (EMRs), prescription systems, and laboratory systems are not always optimal in all NHs, and collaboration with a medical microbiology laboratory consultant is not a standard practice. Moreover, surveillance data on antibiotic use and education on antibiotic-related topics are not regularly available. In addition, the guideline recommends conducting urine cultures in patients displaying signs of tissue invasion, in male patients, in cases of treatment failure, and in instances of recurrent infections (3 to 6 per year) [13]. Based on practical experience, NHs tend to conduct limited culture sampling. Despite the need to improve antibiotic use in NHs, there may be time and budget constraints for applying interventions in this setting.

To investigate the implementation of an AMS program that tailors hospital recommendations to NHs, a project was conducted in one of the largest providers of nursing home care in the South‒East region of the Netherlands. The project group comprised the following members: a medical director and an elderly care physician from the provider of nursing home care, a pharmacist responsible for medication supply, a clinical microbiologist from the medical microbiology laboratory providing diagnostics to the organization, an infection control practitioner offering services to the elderly care organization, a representative from a national committee focused on optimizing antibiotic use (Dutch Working Party on Antibiotic Policy (Dutch acronym is SWAB)), representatives from a national institute working on the development and dissemination of information and solutions for medication use, and an administrative support staff member. During the 14-month project, the AMS approach was formulated, and an A-team was established. Antibiotic treatment protocols for the most common infections in nursing home residents were revised at the regional level, including urinary tract infections (UTIs), lower respiratory tract infections (LRTIs) and skin and soft tissue infections (SSTIs). Scorecards for data collection and assessment were developed to evaluate all antibiotic prescriptions in 4 out of 28 nursing homes. A new rule regarding the use of urine dipstick tests was implemented, and it was required that culture be conducted in accordance with the guideline [13]. A standardized presentation for pharmacotherapy audit meetings (PTAMs) was created to introduce AMS. Additionally, e-learning for nurses and caregivers was developed. A focus group meeting involving residents and their representatives was organized to identify their information needs and preferences on this topic. All aforementioned activities were implemented, evaluated, and adapted as necessary. Finally, physicians were invited to complete a brief questionnaire to evaluate the work of the A-team.

The nursing home facilities met the international definition for nursing homes: ‘A nursing home is a facility with a domestic-styled environment that provides 24-hour functional support and care for persons who require assistance with activities of daily living (ADLs) and who often have complex health needs and increased vulnerability. Residents within a nursing home may stay relatively brief for respite purposes, short term (rehabilitative), or long term, and may also receive palliative/hospice and end-of-life care’ [14]. Moreover, care in Dutch nursing homes is provided by a multidisciplinary team led by an elderly care physician and is publicly funded.

The lessons learned from this project have been compiled into recommendations, which are presented in the current practice guide to help NHs set up an AMS program.

## Conditions for establishing an AMS program

To establish an AMS program, certain basic conditions must be met. The extent to which these conditions are met contributes to the success of the program.

### Recommendations

#### Ensure commitment from the board of directors

One of the crucial conditions is the commitment of the board of directors. This board needs to have a clear vision of the necessity of an AMS program and be willing to allocate the necessary human and financial resources.

#### Ensure that human and financial resources are sufficient to carry out an AMS program that fits the NH

Another essential condition is ensuring that the human and financial resources required for an AMS program are both adequate and appropriate for the NH. Consequently, assign this specific task to a physician. The baseline situation determines the amount of resources needed, which may vary depending on factors such as existing contracts between cooperating parties and the ability to embed AMS topics into regular processes and meetings. Consider establishing service agreements in which AMS is an integral part of the services for a clinical microbiologist, a pharmacist, and an infection control practitioner who are not employees of the organization. Additionally, having up-to-date treatment protocols and written guidelines for appropriate antibiotic prescribing are needed to easily generate overviews of antibiotic prescriptions. Furthermore, ensuring that physicians mention the indication for the antibiotic prescription in the prescription system will reduce the time investment required for the program. If NHs already have high compliance rates regarding antibiotic prescribing, implementing the program will require less effort. Changes in microbiology culture policies may have financial implications. Finally, purchasing education materials and adapting them may require substantial financial investment and time investment from healthcare workers.

#### Form a project team to set up and implement an AMS program

To implement an AMS program, it is crucial to form a project team consisting of professionals with relevant expertise. The team should include, at least, a physician who provides medical care to the residents, a pharmacist who supplies the medication to the NH, and a clinical microbiologist of the medical microbiology laboratory that delivers microbiological diagnostic to the NH and has knowledge on local/regional resistance data. To ensure efficient decision-making and create organisation-wide conditions for the success of an AMS program, it is recommended to appoint a medical director or member of the management team to the project team. If this is not feasible, one of the project team members should be authorized on behalf of the management team to determine the responsibilities of each member of the project team. It is also advisable to appoint a healthcare professional to the project team who is well-versed in the nursing home organisation and has received appropriate training in infection control and antibiotic resistance, such as an infection control practitioner.

## Embedding an AMS program within a nursing home

Overall, a comprehensive approach to embedding an AMS program within a NH creates collaboration with and engagement of relevant stakeholders. It incorporates strategies that support the program’s sustainability and success.

### Recommendations

#### Align antibiotic and Infection control policies and bring both areas of expertise within the responsibility of the same committee

The implementation of an aligned AMS and infection control program and bringing both policy topics under the responsibility of one committee (e.g., infection committee) can help achieve the following objectives: (1) Prevent the development of antibiotic resistance through appropriate use of antibiotics, (2) Detect the presence and transmission of (drug-resistant) bacteria, and (3) Prevent transmission of (drug-resistant) bacteria through hygiene and infection control measures.

#### Set up an antibiotic team (A-team)

Establishing an A-team comprising a physician, pharmacist, and clinical microbiologist can facilitate the development and revision of antibiotic treatment protocols as well as the monitoring of antibiotic use. It is also advised to examine the feasibility of regional collaborations in certain aspects (e.g., development and revision of antibiotic treatment protocols). It can be highly valuable to add an infection control practitioner to the A-team to coordinate the activities. The A-team’s composition allows for a collaborative approach to optimize antibiotic use and promote appropriate prescribing.

#### Discuss the AMS program with all physicians and make the AMS program a regular topic during meetings (e.g., pharmacotherapy audit meetings (PTAMs))

To obtain physicians’ support for the AMS program, they must be involved from the start. Utilizing existing meetings, such as PTAMs or staff meetings, can be an effective way to keep colleagues informed about the program’s progress, A-team activities, and antibiotic treatment protocols. The A-team may also use these meetings to discuss issues related to antibiotic choices in treatment protocols, deviations from protocols, or experiences from previous cases, which could result in topics for additional education. Such education can increase physician competence and willingness to adhere to antibiotic treatment protocols.

#### Offer education on antibiotic use and resistance to nurses and carers

It is recommended to provide education on antibiotic use and antibiotic resistance to nurses and carers for various reasons. These healthcare workers are often the first to recognize signs and symptoms of an infection and serve as the primary contact for residents and their representatives. They play a crucial role in relaying information about residents’ conditions to physicians, who do not see residents every day. In addition, they are responsible for carrying out protocols. For example, protocols depicting the use of infection control measures and measures to reduce the risk of a urinary tract infection (UTI) or (aspiration) pneumonia. Often, they also inform residents/their representatives about antibiotic prescriptions. It is important to discuss the feasibility of different modes of education, such as e-learning, which can reach a large target group with relatively little effort, or group discussion training led by an infection control practitioner. Arrangements should also be made regarding whether healthcare workers will receive training during work or during personal time and whether they will have the opportunity to gain accreditation points.

#### Discuss how volunteers should be informed

In nursing homes, volunteers play an important role in the provision of care, e.g., supporting individual and group activities, assisting caregivers with practical tasks such as serving coffee and tea, aiding in cooking and serving dishes, and offering social and emotional support to residents. This group should be adequately informed about infection control. Specifically, the provision of information on hand hygiene, appropriate measures to take in the event of signs or symptoms of an infection, and food preparation (cooking and serving) should be considered. It is recommended to use reliable, publicly accessible sources of information for the dissemination of general information on infections and antibiotic resistance.

#### Discuss how to inform residents/their representatives

In addition to the fact that residents and their representatives should always be able to consult nurses, carers, or physicians, it is recommended to offer them information through various media such as paper copies (folders/newsletters), audio recordings, and video presentations so that they can absorb the information at their convenience. It is imperative to contact the residents’ council of the nursing home to ascertain their information requirements (for example, information on UTI) and determine the most effective way to convey the information to them. The residents’ council should also support the implementation of an AMS program. Similar to volunteers, it is also recommended for this target group to use reliable, publicly accessible sources of information.

## A-team activities

It is important to define the activities of an A-team, which is responsible for promoting appropriate antibiotic use.

### Recommendations

#### Define the responsibilities and authority of the A-team

It is important to define the responsibilities and authority of the A-team, which include the following: (1) maintaining treatment protocols up-to-date in accordance with (inter)national guidelines, regional resistance data, and culturing policy, and (2) monitoring compliance with treatment protocols based on predetermined selection criteria. Moreover, the A-team derives its authority from its expertise in the field of antibiotics, making its opinion highly valued. In case of deviations from treatment protocols or the A-team’s opinion, prescribing physicians need to state the reason for the deviation in the residents’ record. It is also important to emphasize that although the A-team plays a crucial role in antibiotic management, the ultimate responsibility for prescribing antibiotics remains of the individual physician.

#### Define the working process of the A-team, including selection criteria to identify prescriptions for discussion in the A-team

The working process employed in hospitals will usually not be applicable in NHs. Therefore, it is essential to identify a working process that fits within the NH setting. Based on the project we carried out, we recommend a periodic retrospective review of antibiotic prescriptions for discussion in the A-team. Ideally, in an onsite meeting prior to regularly scheduled plenary meetings (e.g., PTAM). This approach enables A-team members to gain insight into the prescribing behaviour of the preceding months and discuss deviations or issues identified during the plenary meeting. This approach aims to achieve a learning effect for the prescription of antibiotics in the future for all residents. As a consequence, sustainable improvement of the overall quality of antibiotic treatments can be achieved.

To evaluate the activities of the A-team, the following questions could be posed to fellow physicians: (a) How feasible do you consider the treatment protocols to be? (b) If it comes to UTIs, do you think that the nurses adhere to the policy that dipstick tests may only be used after consultation with the physician? (c) What aspects of the A-team’s work process are you satisfied with? (d) Are there any bottlenecks or areas for improvement in the work process of the A-team? (e) Do you agree with the policy regarding culturing? (f) Did you change your prescribing behaviour of antibiotics?

To optimize antibiotic prescriptions in NHs, it is recommended to focus on the most common infections, namely, UTIs, lower respiratory tract infections (LRTIs), and skin- and soft-tissue infections (SSTIs). Selection criteria that could be used to identify prescriptions for discussion in the A-team include: (a) prescriptions lacking an indication in the prescription system; (b) prescriptions lacking a (preliminary) stop date in the prescription system; (c) prescriptions with a duration exceeding 7 days; (d) prescriptions for intramuscular or intravenous antibiotics (if applicable); (e) prescriptions for antibiotics other than the first choice based on the applied treatment protocols; (f) prescriptions for combinations of substances, such as amoxicillin + fluoroquinolone; (g) prescriptions for antibiotics regulated for the treatment of particularly resistant microorganisms; and (h) on request of the prescriber.

To determine the time investment required for A-team activities, it is recommended to analyse the volume of prescriptions over a period of one to two months based on the predetermined selection criteria. Priority may be given to one or more of the predetermined selection criteria depending on the results of the analysis. In the prioritization, factors to consider could include (but are not limited to) the severity and frequency of deviations from the treatment protocols, as well as selection criteria where improvement can be achieved quickly and easily.

We advise to oblige physicians to note the indication for an antibiotic in the prescription system. Prescription systems always offer a free text field that can be used, but often it is possible to add a required field for this information. Adding the indication makes it easier to analyse prescription data in relation to infection types and treatment protocols and saves time for the A-team. In addition, complete and correct registration of kidney function, contraindications, over-the-counter (OTC) medication, intolerances, etc. is essential for pharmacists to intervene when necessary.

#### Define the tasks of all A-team members

**Fig. 1:**
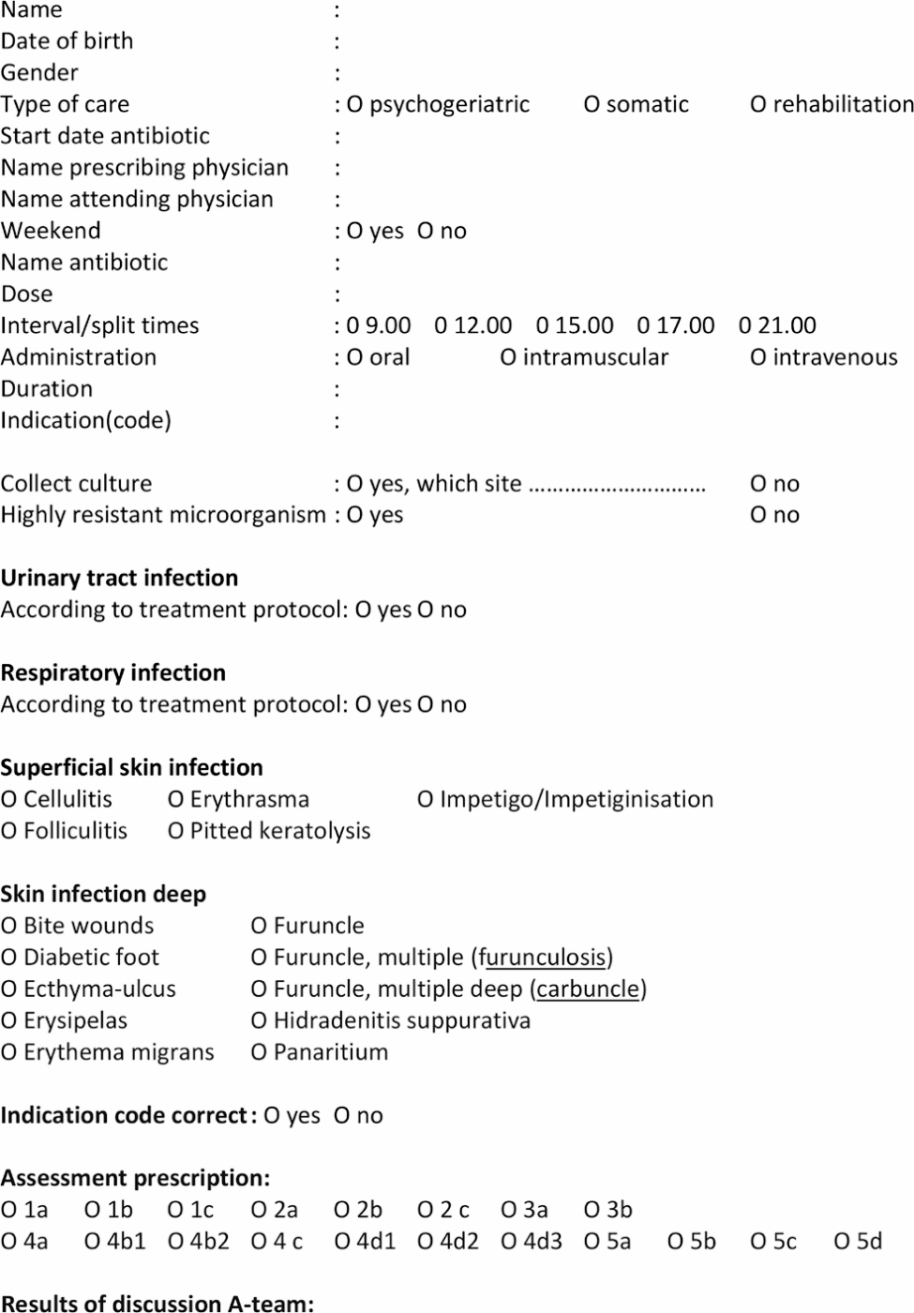
Resident registration form

**Fig. 2:**
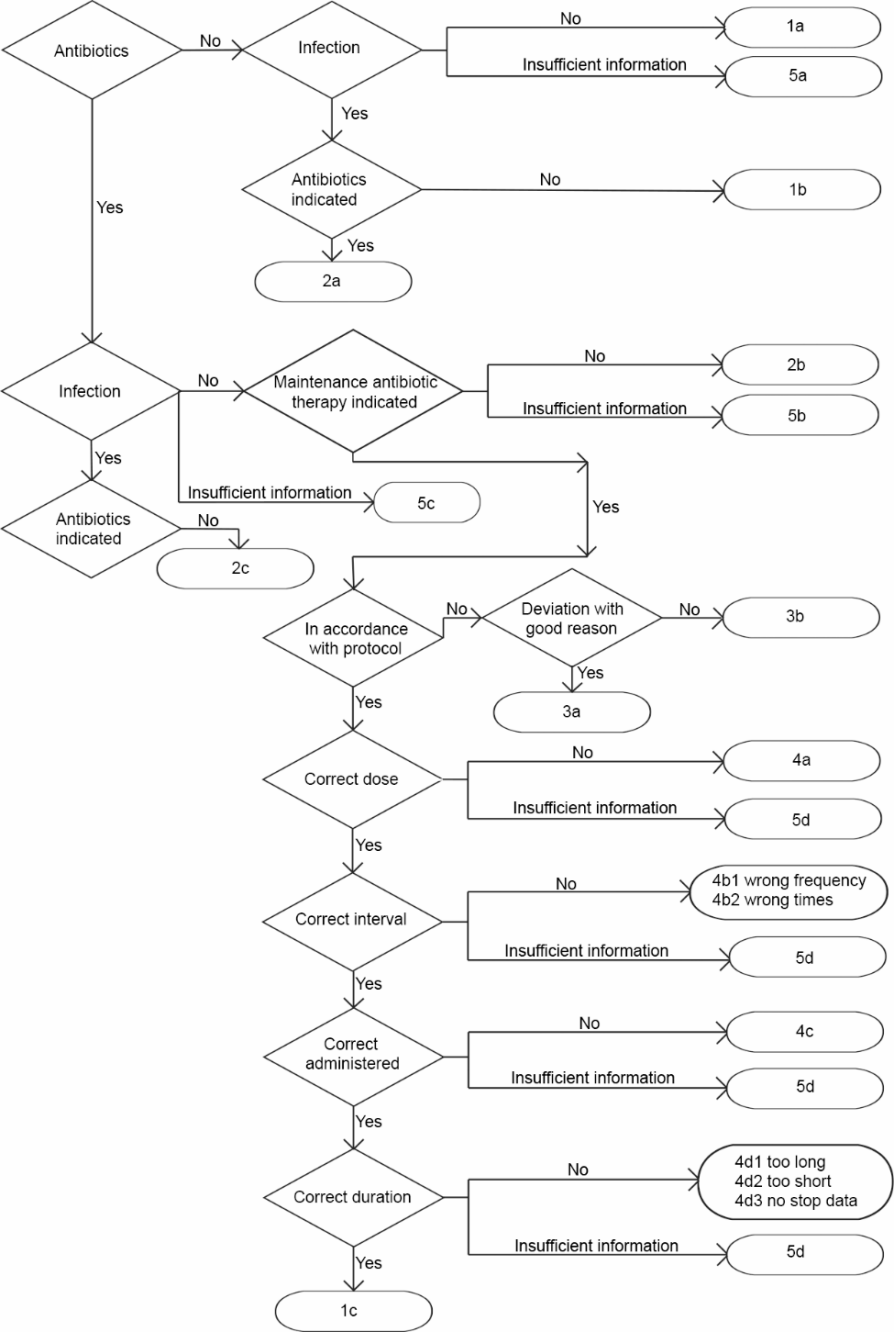
Flowchart of the assessment of antibiotic prescriptions

To ensure optimal functioning of the A-team, it is recommended to define the tasks of each team member. In addition to the A-team’s responsibility for keeping the treatment protocols up-to-date, the following elaboration provides an example of the tasks assigned to each A-team member in monitoring compliance with treatment protocols. The pharmacist generates summaries of antibiotic prescriptions, including the name of the antibiotic, dosage, and duration, based on predetermined criteria. They also review the prescription for potential side effects, toxicity and interactions with other medications used by the resident. The elderly care physician records relevant data from the resident’s medical record on a registration form (see Fig. 1) and requests any missing information from the prescribing physician if necessary. They also provide an assessment regarding the correct or incorrect usage of the antibiotic (see Fig. 2; Table 1). The infection control practitioner collects the registration forms, analyses the data prior to the A-team meeting, and schedules the A-team meetings. Both the elderly care physician and the pharmacist prepare the PTAMs, during which deviations and important issues discussed during the A-team meeting will be addressed. The clinical microbiologist assesses during the A-team meeting whether they agree with the assessment of the antibiotic prescription by the elderly care physician and participates in the PTAM upon request to provide explanations or education on a particular topic.

**Table 1:** Clarification of the flowchart antibiotic prescriptions assessment*

1	Correct decision
1a	No antibiotic(s); no infection; no antibiotic(s) indicated
1b	No antibiotic(s); infection; no antibiotic(s) indicated
1c	Antibiotic(s); infection; antibiotic(s) indicated; in accordance with treatment protocol; correct use
**2**	**Incorrect decision**
2a	No antibiotic(s); infection; antibiotic(s) indicated
2b	Antibiotic(s); no infection; no maintenance therapy indicated; no antibiotic(s) indicated
2c	Antibiotic(s); infection; no antibiotic(s) indicated
**3**	**Incorrect choice of antibiotic(s)**
3a	Antibiotic(s); infection; antibiotic(s) indicated; not in accordance with treatment protocol; deviation with good reason
3b	Antibiotic(s); infection; antibiotic(s) indicated; not in accordance with treatment protocol; deviation without good reason
**4**	**Incorrect use**
4a	Antibiotic(s); infection; antibiotic(s) indicated; in accordance with treatment protocol; wrong dose
4b1	Antibiotic(s); infection; antibiotic(s) indicated; in accordance with treatment protocol; wrong interval: incorrect daily frequency
4b2	Antibiotic(s); infection; antibiotic(s) indicated; in accordance with treatment protocol; wrong interval: incorrect times
4c	Antibiotic(s); infection; antibiotic(s) indicated; in accordance with treatment protocol; wrong administration form (oral/intramuscular/intravenous)
4d1	Antibiotic(s); infection; antibiotic(s) indicated; in accordance with treatment protocol; wrong duration: duration too long
4d2	Antibiotic(s); infection; antibiotic(s) indicated; in accordance with treatment protocol; wrong duration: duration too short
4d3	Antibiotic(s); infection; antibiotic(s) indicated; in accordance with treatment protocol; wrong duration: no stop date
**5**	**Insufficient information**
5a	No antibiotic(s); insufficient information about infection
5b	Antibiotic(s); no infection; insufficient information about maintenance therapy
5c	Antibiotic(s); insufficient information about infection
5d	Antibiotic(s); infection; antibiotic(s) indicated; insufficient information about accuracy of use

*Partly based on the scoring system for the appropriateness of antimicrobial therapy from Willemsen et al. [15] *and adapted to the nursing home setting*.

A summary of the recommendations and their elaboration is included in Table 2.

**Table 2:** Summary of recommendations to implement an AMS program

Basic conditions	Recommendations	Elaboration
1. Conditions for establishing an antimicrobial stewardship (AMS) program	1.1 Ensure commitment from the Board of Directors.	• Define the vision on necessity of an AMS program. • Allocate human and financial resources.
	1.2 Ensure human and financial resources are sufficient to carry out an AMS program that fits the nursing home.	• Allocate a physician for this task. • Establish service agreements in which AMS is an integral part of the services for nonemployed professionals.
	1.3 Form a project team to set up and implement an AMS program.	• Establish a project team comprised of members with relevant expertise: a physician, a pharmacist, a medical microbiologist, an infection control practitioner, a member of the management team (MT) or an authorized project team member, on behalf of the MT.
2. Embedding an AMS program within a nursing home	2.1 Align antibiotic- and infection control policies and bring both areas of expertise within the responsibility of the same committee.	• Consolidate AMS and infection control policies under one committee.
	2.2 Set up an Antibiotic team (A-team).	• Establish an A-team comprised of a physician, pharmacist, clinical microbiologist, and preferably also an infection control practitioner.
	2.3 Discuss the AMS program with all physicians and make the AMS program a regular topic during meetings (e.g., pharmacotherapy audit meetings (PTAMs)).	• Involve all physicians from the start and keeping them informed about the process. • Make the AMS program a regular topic in during meetings (e.g., PTAMs).
	2.4 Offer education on antibiotic use and resistance to nurses and carers.	• Determine the mode(s) of education, as well as whether it should be followed during work or personal time, and the possibility to gain accreditation points.
	2.5 Discuss how volunteers should be informed.	• Use reliable, publicly accessible sources of information to disseminate.
	2.6 Discuss how to inform residents/their representatives.	• Inform residents through various media. • Use reliable, publicly accessible sources of information to disseminate.
3. A-team activities	3.1 Define the responsibilities and authority of the A-team.	• Keep treatment protocols up-to-date. • Monitor compliance. • Emphasize that the opinion of A-team considered as highly valued, although the ultimate responsibility for prescribing antibiotics remains of the individual physician.
	3.2 Define the working process of the A-team, including selection criteria.	• Conduct a periodic retrospective review of the antibiotic prescriptions prior to scheduled plenary meetings (e.g., PTAMs) based on predetermined selection criteria. • Evaluate the activities of the A-team among colleague physicians.
	3.3 Define the tasks of all A-team members.	• Define the tasks of each A-team member.

## Discussion

In this practice guide, we present a feasible approach for NHs to implement an AMS program. This approach is rooted in the ‘Practical Guide Antimicrobial Stewardship in the Netherlands’ [12], which is guided towards hospitals and has been tailored to suit the nursing home setting. This was achieved through close collaboration among experts in the fields of elderly care, antibiotic prescribing, antibiotic resistance, and infection control.

This approach can be adapted to local or regional collaborations between NHs, pharmacies, and clinical microbiologists. With the described working process of the A-team, we aim to achieve a learning effect for future antibiotic prescriptions for all residents, thereby enhancing the overall quality of the prescriptions. Moreover, the presence of a peer-review system in which physicians review their colleagues’ prescribing behaviour is expected to encourage greater attention to prescribing practices.

The hospital practice guide [12] published in 2015 was based on expert opinion and supporting literature. In 2016, the SWAB guideline committee conducted a literature search with the aim of evaluating the quality of evidence for fourteen antimicrobial stewardship objectives in which the LTCF setting was included. At that time, no supporting evidence for LTCF was found, nor was any contradictory evidence found [16]. Meanwhile, several guidelines have been provided on the implementation of an AMS program in LTCFs [17–19] based on seven core elements published by the Centers for Disease Control and Prevention in 2015 [20]. These seven core elements described are derived from an adaptation of the elements described for hospital antibiotic stewardship and are supported by reviews, intervention studies, regulations, consensus, and surveillance data. No quality assessment of the included studies took place, and the studies were not graded. This also applies to the aforementioned guidance papers [17–19].

In recent years, several systematic reviews have collectively indicated that the implementation of an AMS program has the potential to optimize antimicrobial use in LTCFs [21–24]. The recommendations in our practice guide are corroborated by these studies; however, caution is warranted. The reviewed AMS programs are all unique, lacking standardization in terminology, strategy, evaluation, or reporting. Nevertheless, we were able to compare the strategies in broad terms. Considering the recommendations related to conditions for establishing an AMS program in our practice guide, the recommendation to ensure commitment from the board of directors is not explicitly described in any of the included studies in these reviews. Although it may be considered implicit, we assert the necessity of explicitly stating this, as outlined in the hospital practice guide [12]. Ensuring that human and financial resources are sufficient to carry out an AMS program tailored to nursing homes is outlined in the review from Wu et al. [21]. Establishing a project team to set up and implement an AMS program is a demonstrated approach across all these reviews [21–24]. However, the elaboration of this recommendation varies across studies. Turning to the recommendations pertaining to embedding an AMS program within a nursing home, aligning antibiotic and infection control policies and bringing both areas of expertise within the responsibility of the same committee, as also described by the SWAB [25], is advocated by the review of Katz [24]. The establishment of an A-team is demonstrated in all these reviews [21–24], albeit the elaborations also vary among the studies. The same applies to the recommendation to discuss the AMS program with all physicians and make it a regular topic during meetings. Offering education on antibiotic use and resistance to nursers and carers is collaborated by Wu et al., Raban et al., and Crespo-Rivas et al. [21–23]. None of the reviews included studies on information for volunteers, as we have recommended. We emphasized the importance of this recommendation due to the role volunteers play in the provided care within Dutch nursing homes. Offering information for residents and their representatives is also supported by Wu et al. Raban et al. and Crespo-Rivas et al. [21–23]. Regarding the recommendations related to the A-team activities, recommendations defining the responsibilities and authority of the A-team, the working process of the A-team, and the tasks of all A-team members are corroborated by all reviews; however, here as well, the elaboration also varies in the studies. In addition to referencing the four reviews, it is noteworthy to mention the study by Stone et al. [26], which supports our recommendation to appoint an infection control practitioner in both the project team and A-team. The study revealed a significant positive association between NHs having trained infection control practitioners and performing stewardship activities.

Another crucial aspect to be noted is that in the USA, it became mandatory by law to integrate AMS into infection control programs in NHs in 2016. A survey conducted in NHs showed that the implementation of all seven core elements increased from 43% to 2016 [27] to 71% in 2018 [28]. However, the implementation of an AMS program is still not mandatory in European LTCFs. The results of a survey conducted among LTCFs in Europe [29] regarding the presence of AMS based on ten elements in 2016 and 2017 showed large variation between the participating countries and the ten elements. It is noteworthy that more than half of the LTCFs lacked a therapeutic formulary and written guidelines for appropriate antimicrobial use, which are the basis for the rational, appropriate, and safe use of antibiotics. As far as we know, recent data on the extent to which stewardship activities have increased in European LTCFs are lacking.

In general, it is crucial to facilitate NHs in implementing an AMS program. NHs should have the flexibility to choose an approach that aligns with their organizational structure. Our current practice guide offers practical tools for establishing an AMS program, which can be considered separate parts of a toolbox. The local context determines the most effective way to utilize the A-team and implement the AMS program’s tools. The involved physician should play a significant role in creating a sense of urgency, prioritizing program elements, and proposing a step-by-step approach. In the Netherlands, the Dutch Association of Elderly Care Physicians has endorsed the role of elderly care physicians in infection control and antibiotic resistance in its general guideline [30].

Given the lessons learned from our project, we recommend retrospectively reviewing antibiotic prescriptions by the A-team until EMR, pharmacy, and laboratory systems are appropriately configured to enable automatic feedback upon an antibiotic prescription at the individual level of residents. Subsequently, the results of these reviews should be periodically discussed during regular meetings, such as PTAMs. This approach has two limitations. First, this approach precludes the possibility of individual client adjustments. Second, our focus is limited to residents who receive antibiotics, whereas those who do not receive antibiotics, even when it may be indicated, are excluded. Therefore, we recommend conducting repeated prevalence studies on antibiotic use among all residents.

In conclusion, the development of tailored AMS programs that are feasible in NHs can be facilitated by collaborative efforts between physicians, pharmacists, clinical microbiologists and infection control practitioners, preferably on a regional level.

## Data Availability

Not applicable.
